# Correspondence

**Published:** 1900-07

**Authors:** C. B. Wright


WEDOWEE, ALA.

*Editor Atlanta Journal-Record of Medicine:*

DEAR SIR—I take pleasure in reporting to your valuable journal the following case:

The patient was a multipara, having given birth to six children, four boys and two girls, prior to the following labor. All well developed and with normal labors. History of parents good except a light case of scrofula in mother when very small. Fetus carried to full term, 280 days, imperfectly developed, with a mere sign of penis and scrotum, just enough to reveal sex. Fetus delivered breech foremost, with scarcely any labor pains. Placenta imperfectly developed. Cord degenerated from stenosis at the umbilical origin. Fetus weighed two pounds. Mother a white woman, aged 30. Very truly,

C. B. WRIGHT, M.D.

---

### TREATMENT OF CONVULSIONS IN CHILDREN.

Kings uses the following in cases of convulsions in infants under six months of age, whether due to dentition, falls on the head, meningitis, or other causes:

℞ Calcium sulphate .................................. 0.06 gm.
Distilled water .................................. 250.00 gm.
M. S.—A teaspoonful every hour. (To be freshly prepared.)
*Bull. Gén. de Thér.*, February 28, 1900.

